# Social Data: An Underutilized Metric for Determining Participation in COVID-19 Vaccinations

**DOI:** 10.7759/cureus.16379

**Published:** 2021-07-14

**Authors:** Alec D McCarthy, Daniel J McGoldrick, Phil A Holubeck, Cavan Cohoes, Laura D Bilek

**Affiliations:** 1 Department of Surgery - Transplant, University of Nebraska Medical Center, Omaha, USA; 2 Department of Computer Science, California State University, Monterey Bay, Seaside, USA; 3 Department of Regenerative Medicine, College of Medicine, University of Nebraska Medical Center, Omaha, USA; 4 Department of Mathematics, Columbia University, New York, USA; 5 College of Allied Health Professionals, University of Nebraska Medical Center, Omaha, USA

**Keywords:** johnson and johnson vaccine, vaccine hesitancy, covid-19 vaccination, vaccination hesitancy, social data, google trends, vaccine participation, vaccine, moderna vaccine, pfizer vaccine

## Abstract

Many measures have been taken since late 2019 to combat the coronavirus disease (COVID-19) pandemic. National, state, and local governments employed precautions, including mask mandates, stay-at-home orders, and social distancing policies, to alleviate the burden on healthcare workers and slow the spread of the severe acute respiratory syndrome coronavirus 2(SARS-CoV-2) virus until an efficacious vaccine was made widely available. By early spring of 2021, three effective and well-tolerated SARS-CoV-2 vaccines emerged and underwent broad distribution. Throughout the course of the COVID-19 vaccination campaign, several key logistical and psychological issues surfaced. Of these, access to vaccines and vaccination hesitancy are cited as two substantial hindrances towards vaccination. Noting the demand for the SARS-CoV-2 vaccine and its highly sensitive storage requirements, accurate dose allocation is critical for vaccinating the population quickly and successfully. Here, we propose the use of social data as a tool to predict vaccination participation by correlating Google searches with state-level daily vaccination. We identified a temporal and regionally-ubiquitous Google search syntax that broadly captures daily vaccination trends. By correlating trends in the search syntax with daily vaccination rates, we were able to quantify the correlation and identify optimal lag periods between Google searches and daily vaccination. This work highlights the importance of analyzing social data as a metric to effectively arrange vaccination roll-outs, identify voluntary vaccination participation, and identify inflection points in vaccination participation. In addition, social data assessments can help direct dose allocation, identify geographic areas that may seek, but lack, access to the vaccines, and actively prepare for fluctuations in vaccination demands.

## Introduction

Since the emergence of the severe acute respiratory syndrome coronavirus 2 (SARS-CoV-2) virus in late 2019, global efforts towards slowing the spread of the virus have involved implementing mask mandates, social distancing guidelines, and work-from-home policies, all of which vary at the city, county, and state levels [1-5]. The aim of each measure was to slow the spread of the virus until an efficacious vaccine emerged. By early fall of 2020, Moderna (Cambridge, Massachusetts), Pfizer/BioNTech (Pfizer, Manhattan, New York City), and Janssen/Johnson & Johnson (JNJ; Beerse, Belgium) emerged as the three primary vaccine manufacturers with leading vaccine candidates [6]. The US Food and Drug Administration (FDA) granted all three vaccines emergency authorization after it was determined they provided immunity against a SARS-CoV-2 infection.

The US Center for Disease Control and Prevention (CDC) has since led efforts to vaccinate as many people as possible, utilizing federal, state, county, and city vaccination clinics [7-9]. Several key factors have thus far determined the success of these vaccination efforts. First, logistical hurdles (i.e., proximity to clinics, access to vaccines) present challenges to many populations in rural areas [10]. Second, psychological hurdles (i.e., fear of side effects, preference in vaccine manufacturer, distrust in vaccine platforms) may also contribute to hesitancy in voluntary vaccine participation [11-13]. The CDC’s Vaccine Tracking System (VTrckS) publishes daily updates on the vaccine effort that includes daily vaccinations, cumulative vaccinations, doses utilized, and numerous other metrics [14]. Doses have been allocated on state, city, and county levels based on a variety of criteria. However, one seldom-mentioned method to allocate vaccines and analyze voluntary vaccination participation is social data. To rapidly assess temporal and regional social interest in a given topic or search phrase, Google Trends (GT) can easily be employed [15].

GT is a public feature of Google that summarizes temporal and regional search query data presented as relative search volume (RSV). RSV is recorded daily, updated instantaneously, easy to access, and offers the ability to download a query’s raw data [16-17]. In fact, a variety of studies have used GT data to predict emerging COVID-19 cases, assess participation in a variety of medical procedures, and map vaccination hesitancy [15,18-21]. Noting that Google is the most widely-used search engine, we hypothesized that temporal and regional trends in vaccine-related search phrases would significantly correlate with realized vaccination participation [22]. Further, we sought to investigate temporal and regional vaccination sentiment by analyzing both positive and negative interest in manufacturer-specific search queries. Due to many states requiring appointments in order for their citizens to receive a vaccination, we hypothesized that there would be a lag in vaccination data when compared to RSV. By checking cross-correlation coefficients, we were able to identify states with significant increases in correlation as a result of the lag, indicating the possible strengths and limitations of vaccination roll-out protocols. Finally, we sought to establish an optimal lag time to validate the use of social data as a tool in determining vaccination participation and dose allocation.

## Materials and methods

Surveying public interest with Google Trends

GT is a publicly available online search engine available from Google that generates aggregate search data over a user-specified set of criteria. It provides users a largely unfiltered, real-time look at trending RSVs across any terms or temporal windows supplied [23]. These terms can be compiled to cover a large swathe of related queries with the intent to identify a larger picture or trend. The terms can also be bundled separately as a method to track the relationships between aggregate volumes and search popularity. Furthermore, GT supplies users with the ability to track RSV (normalized on a 0-100 scale) based on a specific city, state, country, or as a worldwide trend. Output data from GT result in temporal and geographical (including country, state, county, and city-level granularity) RSVs that are normalized by population. Users can select and examine specific time windows dating back to 2004. The most granular time interval available is the hourly aggregate, with the longest window available being monthly. Notably, GT is not case sensitive (i.e., searching "*covid*" and "*COVID*" generates the same results). However, GT is character-sensitive such that exact phrasing is relevant in generated RSVs (i.e., searching "*COVID19*" and "*COVID-19*" generate different results.

Determination of temporal and regional syntax sensitivity

Prior to any correlative analyses, it was critical to determine what search phrase had the highest average temporal RSV and highest regional RSV. Identifying the most ubiquitous search phrase associated with the COVID-19 vaccination efforts allows us to most accurately encapsulate search interest across all states (including Washington D.C. (DC)) between October 1, 2020, and April 9, 2021, a temporal window that begins with the onset of measurable RSV in obtaining the covid vaccine. After defining our timeline of interest, GT-procured related search terms were analyzed for the duration of relatively high/low RSV, average RSV over time, and average regional RSV throughout the predetermined time frame. To determine the most sensitive syntax, we followed GT's prompt to look at coronavirus search trends on its "Featured" tab. From this prompt, “*covid vaccine near me*” was a trending search. The remaining search queries “*covid vaccine appointment*,” “*where to get covid vaccine*,” and “*how to get covid vaccine*” were related searches automatically suggested by GT. An aggregate search with all four terms was conducted and the temporal, average temporal, and regional data were generated and compared.

Determination of public sentiment toward different vaccine manufacturers 

To investigate public interest in each vaccine, we conducted aggregate temporal and regional searches with the names of the three largest COVID-19 vaccine manufacturers. Interest in the search phrases “Moderna vaccine,” “Pfizer vaccine,” and “Johnson and Johnson vaccine” was examined from October 1, 2020, to April 9, 2021. Other manufacturers (i.e., Inovio, Novavax, AstraZeneca, Sinovac, etc.) were excluded from the aggregate search, as their RSVs were too small for GT to quantify. Each manufacturer’s temporal and regional RSV were plotted and compared. In the context of these vaccination efforts, it is advantageous to consider sentiment with search volume. For example, in some cases, search interest may be tied with negative sentiments that would be counterproductive to consider as support for vaccination participation. To gain insight on public sentiment towards each vaccine manufacturer, a second search of each vaccine manufacturer’s name with the addition of “side effects” was conducted (i.e., "Moderna vaccine side effects"). As before, each search term’s temporal and regional RSV was plotted and compared. The inclusion of a search observing sentiment was included to identify discrepancies in search interest of manufacturer-specific vaccine-related side effects compared to interest in the vaccine alone.

Determination of state population, daily vaccinations, and dose utilization

To determine if population impacted RSV in vaccine-related search phrases, the Vintage 2020 population estimate data from the United States Census Bureau (USCB) was utilized. The USCB uses a year-over-year revision system to update annual estimates such that the estimated predictions are as accurate to census results as possible. Data from the USCB was used as a visual reference to discern population-dependent trends. Vaccination data were accessed from the United States Center for Disease Control and Prevention (CDC), which is updated daily on a county, state, and national level. The CDC reports daily vaccinations as new doses administered per day (7-day smoothed). In cases where data is not reported daily, the CDC assumes doses changed equally on a daily basis over the periods in which data was not reported. Dose utilization is defined as the fraction of vaccine doses (including first and second doses) administered among the quantity of doses recorded as shipped by the CDC’s Vaccine Tracking System. In context, it is difficult to determine whether doses were wasted (i.e., failure to show up for an appointment) or unused (i.e., in storage waiting for administration). In its most basic sense, dose utilization can be thought of as the ratio of administered vaccine doses to total doses in a state’s possession. All of the data sets utilized in this study are publicly available and easily accessible for constant model updating.

Determining correlation and lag between RSV and vaccine participation

In order to determine any correlation between RSV and daily vaccination, we transformed daily vaccine numbers into a percent-of-max scale. This allowed the data to be compared directly to RSV, which is already normalized on a percent-of-max scale. To determine the correlation between the two lines, a linear association was measured using simple linear regression and determination of Pearson’s product-moment correlation coefficient (Pearson’s correlation), given as a measure of linear association between two variables. To determine the optimal lag, cross-correlation coefficients were computed to elucidate lines of best fit for their respective slopes. In this case, the value of the lag with the highest correlation coefficient represents the optimal lag.

## Results

Determination of temporal and regional syntax sensitivity

**Figure 1:**
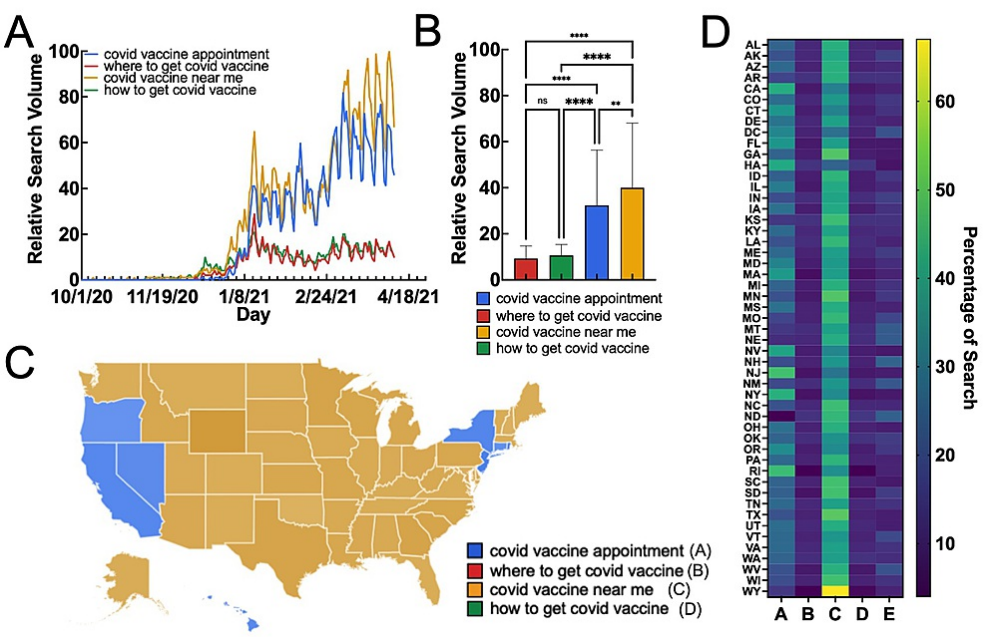
Temporal and regional determination of highest sensitivity syntax (A) Temporal RSV of searches relating to participation in COVID-19 vaccination. (B) Average RSV of each search term over the given time period. (C) US map illustrating the highest state average RSV for each term. (D) Individual state RSV for each search phrase (A = covid vaccine appointment, B = where to get covid vaccine, C = covid vaccine near me, D = how to get covid vaccine). RSV: relative search volume Data source: Google Trends

Syntax is extremely important when considering and identifying trends, as certain search phrases are more effective in accurately and ubiquitously capturing broader search trends on temporal and regional metrics. From October 1, 2020, to April 9, 2021, the phrase “*covid vaccine near me*” had an average RSV of 40.01 ± 28.12, “*covid vaccine appointment*” had an average RSV of 32.34 ± 23.97, “how* to get covid vaccine*” had an average RSV of 10.62 ± 4.73, and “*where to get covid vaccine*” had an average RSV of 9.25 ± 5.51 (Table 1, Figure 1A). The search term “*covid vaccine near me*” had a significantly higher RSV than each of the other terms, indicating the search phrase was the most frequently searched over the time interval (Figure 1B). Similarly, the regional data indicated the two search terms “*covid vaccine near me*” and “*covid vaccine appointment*” were the most geographically-encompassing search terms, with “*covid vaccine near me*” having the highest RSV in 42 states (darker shades of each color corresponding to a higher RSV) (Figure 1C). Summary data for each states’ relative searches is presented in Figure 1D and Table 2. Notably, the states that had “covid vaccine appointment” as the highest search phrase included California (CA), Oregon (OR), Nevada (NV), Hawaii (HI), New York (NY), New Jersey (NJ), Rhode Island (RI), and Connecticut (CT). Overall, considering the temporal and regional results, the search term most correlated with daily vaccines was “*covid vaccine near me*”, which was used in daily vaccination correlation analysis.

**Table 1:** Summary RSV data of sensitivity syntax RSV: relative search volume

	Search Terms				
	covid vaccine appointment	where to get covid vaccine	covid vaccine near me	where to get covid vaccine	how to get covid vaccine
Sum	4204	1202	5221	1202	1399
Average RSV	32.34	9.25	40.01	9.25	10.62
Standard Deviation	23.97	5.51	28.12	5.51	4.73

**Table 2:** State-level RSV of search phrases related to COVID-19 vaccine participation RCV: relative search volume

State	Search Term				
	COVID vaccine appointment	where to get COVID vaccine	COVID vaccine near me	where to get COVID vaccine	how to get COVID vaccine
Alabama	24%	11%	44%	11%	10%
Alaska	20%	11%	42%	11%	16%
Arizona	25%	10%	44%	10%	11%
Arkansas	17%	13%	44%	13%	13%
California	43%	8%	31%	8%	10%
Colorado	26%	11%	38%	11%	14%
Connecticut	37%	9%	32%	9%	13%
Delaware	28%	11%	40%	11%	10%
District of Columbia	33%	10%	27%	10%	20%
Florida	37%	8%	39%	8%	8%
Georgia	24%	9%	50%	9%	8%
Hawaii	42%	13%	24%	14%	7%
Idaho	23%	9%	45%	9%	14%
Illinois	30%	8%	42%	9%	11%
Indiana	19%	12%	41%	12%	16%
Iowa	27%	9%	42%	9%	13%
Kansas	14%	13%	47%	13%	13%
Kentucky	25%	11%	41%	11%	12%
Louisiana	20%	12%	46%	12%	10%
Maine	25%	10%	39%	11%	15%
Maryland	32%	9%	38%	9%	12%
Massachusetts	38%	6%	39%	7%	10%
Michigan	24%	9%	44%	10%	13%
Minnesota	20%	8%	50%	8%	14%
Mississippi	27%	10%	43%	10%	10%
Missouri	17%	10%	46%	10%	17%
Montana	14%	12%	40%	12%	22%
Nebraska	12%	11%	43%	12%	22%
Nevada	41%	9%	33%	9%	8%
New Hampshire	19%	10%	37%	10%	24%
New Jersey	48%	7%	28%	8%	9%
New Mexico	18%	12%	37%	12%	21%
New York	44%	7%	34%	7%	8%
North Carolina	20%	11%	47%	11%	11%
North Dakota	4%	13%	46%	14%	23%
Ohio	27%	10%	44%	10%	9%
Oklahoma	24%	12%	36%	13%	15%
Oregon	34%	9%	34%	9%	14%
Pennsylvania	25%	10%	45%	10%	10%
Rhode Island	47%	4%	35%	4%	10%
South Carolina	24%	10%	48%	10%	8%
South Dakota	23%	7%	48%	7%	15%
Tennessee	25%	11%	40%	11%	13%
Texas	20%	10%	51%	10%	9%
Utah	26%	11%	43%	11%	9%
Vermont	25%	10%	37%	10%	18%
Virginia	25%	11%	40%	11%	13%
Washington	24%	12%	39%	13%	12%
West Virginia	20%	8%	44%	8%	20%
Wisconsin	19%	10%	48%	10%	13%
Wyoming	14%	4%	67%	5%	10%

Public interest and sentiment for different vaccine manufacturers

**Figure 2:**
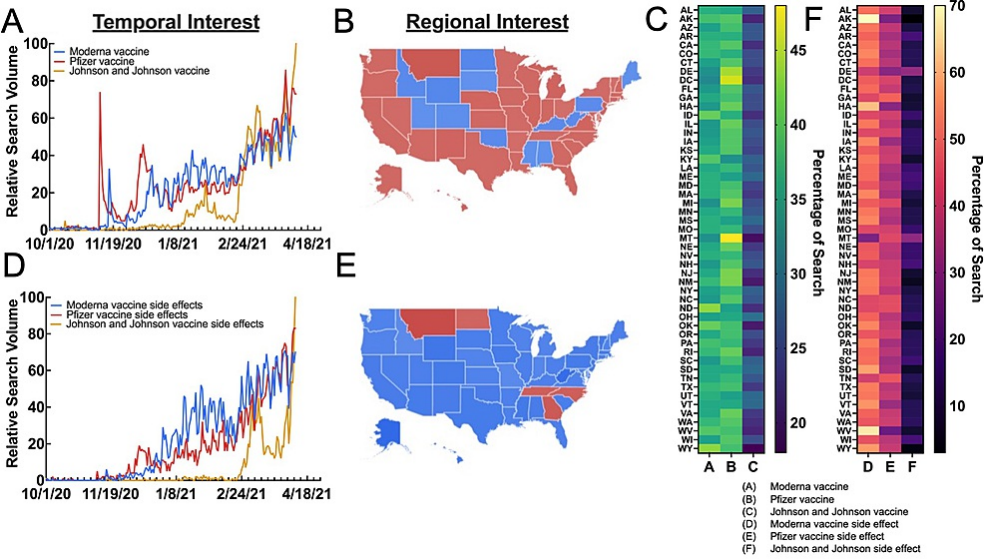
Public interest and sentiment in vaccination by manufacturers (A) Temporal and (B) regional interest in the three major COVID-19 vaccine manufacturers. (C) State interest in each of the three major COVID-19 manufacturers. (D) Temporal and (E) regional interest in each vaccine manufacturer’s side effects. (F) State interest in each vaccine manufacturer’s side effects Data source: Google Trends

Initially, interest in the Moderna and Pfizer vaccine prevailed over JNJ, though search interest for the three approached insignificant differences by late February, corresponding with the U.S. Food and Drug Administration's approval of the JNJ vaccine. RSV for “Pfizer vaccine” summed 4601, averaging 24.09 ± 20.02 from October 1st, 2020 through April 9th, 2021, while “Moderna vaccine” summed 4260, averaging 22.30 ± 17.83, and “Johnson and Johnson vaccine” summed 2830, averaging 14.82 ± 22.46 over the same time period (Figure 2, panel A). Interest in manufacturer-specific vaccines showed some regional preference, with Idaho (ID), Colorado (CO), Utah (UT), Wyoming (WY), North Dakota (ND), South Dakota (SD), and Oklahoma (OK) (central west and upper midwest); Alabama (AL), Mississippi (MS), Kentucky (KY), and West Virginia (WV) (southeast), and Pennsylvania (PA) and Maine (ME) having the highest interest in “Moderna vaccine” and the remainder of states having the highest interest in “Pfizer vaccine” (Figure 2, panel B). Each state's RSV for each vaccine manufacturer is summarized in a heat map in Figure 2, panel C. 

To analyze sentiment regarding vaccine side effects, a similar query searching “Moderna vaccine side effects,” “Pfizer vaccine side effects,” and “Johnson and Johnson vaccine side effects” was carried out over the aforementioned time period. In this case, one would assume search interest for vaccine side effects would likely correspond to news-catalyzed events such as the hypothetical discontinuation of a vaccine. However, the RSVs for each vaccine manufacturer’s side effects showed a clear pattern. Interest in “Moderna vaccine side effects" had an average RSV of 22.60 ± 22.54, with “Pfizer vaccine side effects” and “Johnson and Johnson vaccine side effects” having RSVs of 18.03 ± 19.76 and 5.23 ± 10.99, respectively (Figure 2, panel D). Notably, however, was the immediate and rapid ascension of interest in “Johnson and Johnson vaccine side effects” in early April 2021, which coincided with reports of JNJ’s vaccine causing cerebral venous sinus thrombosis (CVST). Regionally, every state besides Montana (MT), North Dakota (ND), Tennessee (TN), North Carolina (NC), and Georgia (GA) (which all had the highest interest in “Pfizer vaccine side effects”), had the highest RSV for “Moderna vaccine side effects” (Figure 2, panel E). Based on sentiment data, it is apparent that most of the interest in vaccine side effects are related to Moderna’s vaccine. State-wide summary data for interest in each manufacturer’s side effects are presented as a heat map in Figure 2, panel F.

Dose utilization 

**Figure 3:**
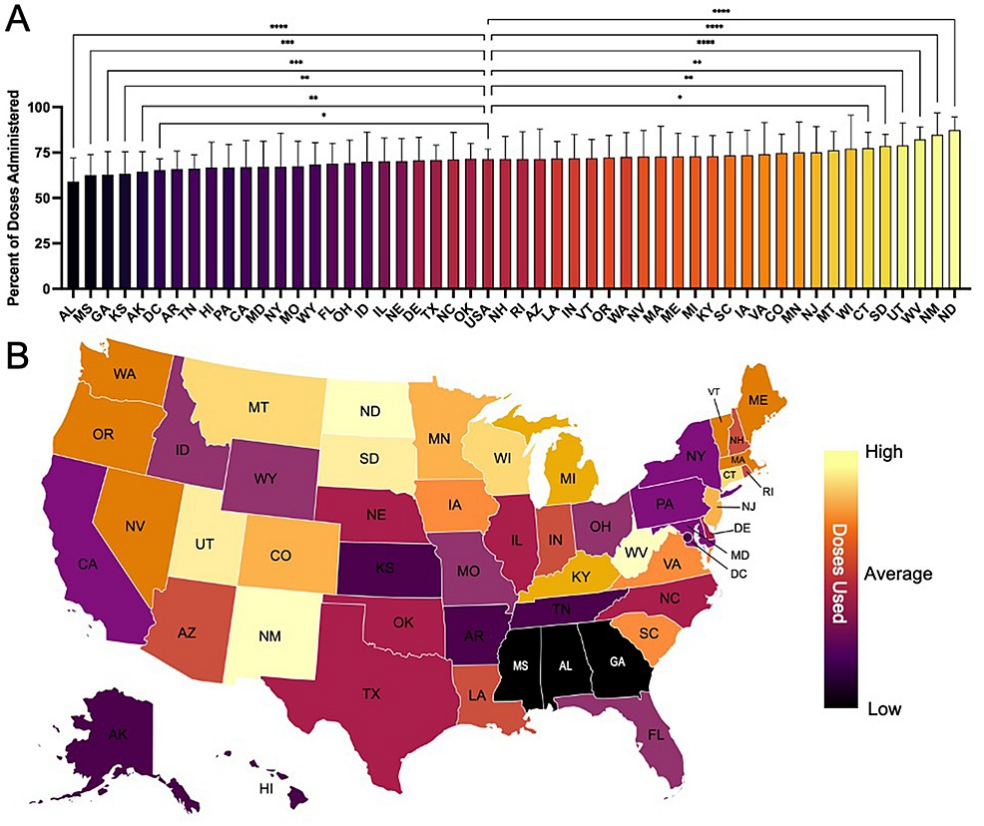
State-level vaccine dose utilization (A) Percent of received COVID-19 vaccine doses administered. (B) Geographic heat map illustrating regional differences in dose utilization (dark = low utilization (50%); light = high utilization (95%)) Source: CDC VTrckS

A summary of dose utilization is presented in Figure 3A. Interestingly, dose utilization showed a strong regional trend, with the lowest dose utilization generally occurring in the Southeast (SE) and the highest occurring in the upper Midwest (MW) (Figure 3B). The states with dose utilization significantly lower than the national average (71.43%) were Washington DC (DC) (65.26%), Alaska (AK) (64.48%), Kansas (KS) (63.4%), Georgia (GA) (62.76%), Mississippi (MS) (62.57%), and Alabama (AL) (59.06%). Conversely, the states with significantly higher dose utilization compared to the national average were Connecticut (CT) (77.54%), South Dakota (SD) (78.75%), Utah (UT) (78.84%), West Virginia (WV) (82.25%), New Mexico (NM) (84.85%), and North Dakota (ND) (87.38%). 

Determining correlation and lag between RSV and vaccine participation

**Figure 4:**
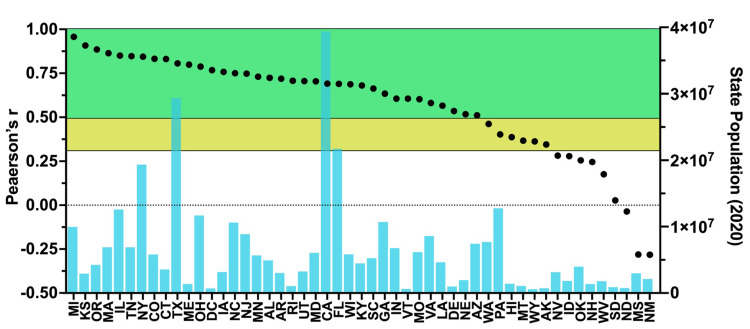
Optimal lag correlation (individual dots) and population (aqua blue bars) of each state White graph area = no correlation/negative correlation; yellow graph area = moderate correlation; green graph area = strong correlation) Data source: Google Trends and United States Census Bureau

**Figure 5:**
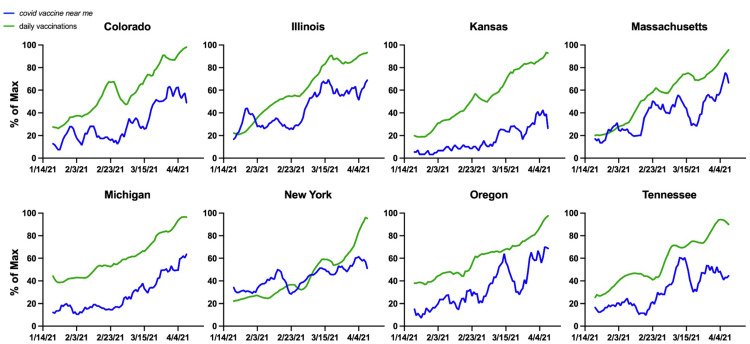
States with highest correlation values between RSV for “covid vaccine near me” and daily vaccinations, both expressed relative to their maximum over the given time period RSV: relative search volume Data source: Google Trends and CDC VTrckS

When determining the impacts of RSV on vaccination efforts, it is important to consider the non-instantaneous nature of our appointment-based system. By adjusting the time series, we can observe the relationship between RSV, state-level vaccination data, and time. To illustrate some optimal predictive relationships, eight states with the highest correlations values were charted in full in Figure 4. We set the window of possible lags to a maximum 14-day temporal window. After setting the spectrum of possible lag values, we measured the direct relationship between the normalized RSV of "*covid vaccine near me*" to the CDC’s normalized data on daily vaccinations per million. Taking the optimal cross-correlation value and observing results between states yielded net improvements in correlative values for many states, with 42 states having a Pearson's r > 0.3 (considered moderately correlated) and 36 states having a Pearson’s r > 0.5 (considered strongly correlated) (Figure 4) [24]. Figure 5 shows eight states (CO, IL, KS, MA, MI, NY, OR, TN) with highly correlated RSV and daily vaccination plots and illustrates the concept of a spike in RSV translating to a spike in realized daily vaccination numbers. Notably, only nine states (NV, ID, OK, NH, WV, SD, ND, MS, NM) had no significant correlation between RSV and daily vaccinations. States with insignificant correlations had smaller populations, perhaps leading to low RSV despite the steps GT takes to normalize population-dense data. Detailed individual state correlation graphs reveal varying degrees of predictive accuracy and are given in the Appendices and Table 3.

**Table 3:** RSV to daily vaccination correlation values with estimated 2020 state population RSV: relative search volume

State	Pearson's R	Population
Alabama	0.724	9966555
Alaska	0.345	2913805
Arizona	0.511	4241507
Arkansas	0.719	6893574
California	0.691	12587530
Colorado	0.832	6886834
Connecticut	0.831	19336776
Washington D.C.	0.768	5807719
Delaware	0.535	3557006
Florida	0.69	29360759
Georgia	0.634	1350141
Hawaii	0.387	11693217
Idaho	0.279	712816
Illinois	0.85	3163561
Indiana	0.606	10600823
Iowa	0.758	8882371
Kansas	0.908	5657342
Kentucky	0.68	4921532
Louisiana	0.566	3030522
Maine	0.799	1057125
Maryland	0.704	3249879
Massachusetts	0.864	6055802
Michigan	0.957	39368078
Minnesota	0.73	21733312
Mississippi	-0.281	5832655
Missouri	0.603	4477251
Montana	0.367	5218040
Nebraska	0.517	10710017
Nevada	0.282	6754953
New Hampshire	0.246	623347
New Jersey	0.748	6151548
New Mexico	-0.282	8590563
New York	0.844	4645318
North Carolina	0.75	986809
North Dakota	-0.036	1937552
Ohio	0.788	7421401
Oklahoma	0.255	7693612
Oregon	0.885	12783254
Pennsylvania	0.403	1407006
Rhode Island	0.707	1080577
South Carolina	0.663	582328
South Dakota	0.027	731158
Tennessee	0.847	3138259
Texas	0.806	1826913
Utah	0.705	3980783
Vermont	0.606	1366275
Virginia	0.581	1784787
Washington	0.462	892717
West Virginia	0.176	765309
Wisconsin	0.687	2966786
Wyoming	0.363	2106319

Observing all states as a population and taking the optimal lag across the possible 14-day window yielded a mean-relative percentage improvement in r2 of 9.90% (95 %CI 5.27% - 14.5%) with the most optimal lag being 4.55 days (95% CI 5.78 - 3.31). States that showed significantly increased goodness-of-fit included Alaska (AK), California (CA), Georgia (GA), Kentucky (KY), Louisiana (LA), Montana (MT), New Hampshire (NH), Pennsylvania (PA), Virginia (VA), and West Virginia (WV), of which a mean improvement among outliers was 37.2% and a mean optimal lag of 9 days or (95% CI 11.7 - 6.50) (Table 4). 

**Table 4:** Optimal-lag adjusted RSV to daily vaccination correlation values RSV: relative search volume

State	Optimal Lag Time (days)	Adjusted Pearson’s R
Alabama	10	0.775
Alaska	8	0.5369
Arizona	5	0.5301
Arkansas	1	0.7021
California	11	0.8106
Colorado	5	0.858
Connecticut	6	0.892
Washington D.C.	11	0.7963
Delaware	2	0.5546
Florida	1	0.6911
Georgia	8	0.7925
Hawaii	14	0.3637
Idaho	8	0.3104
Illinois	4	0.8599
Indiana	1	0.5801
Iowa	1	0.7391
Kansas	8	0.9267
Kentucky	10	0.7831
Louisiana	3	0.6526
Maine	6	0.897
Maryland	5	0.7582
Massachusetts	1	0.8636
Michigan	1	0.9528
Minnesota	1	0.7181
Mississippi	1	-0.219
Missouri	3	0.6058
Montana	14	0.5819
Nebraska	1	0.5119
Nevada	1	0.2749
New Hampshire	4	0.4127
New Jersey	9	0.8298
New Mexico	1	-0.3477
New York	4	0.8781
North Carolina	7	0.7709
North Dakota	1	-0.055
Ohio	3	0.7952
Oklahoma	1	0.2386
Oregon	1	0.8806
Pennsylvania	14	0.6141
Rhode Island	13	0.756
South Carolina	2	0.6774
South Dakota	1	0.0152
Tennessee	5	0.8875
Texas	3	0.8725
Utah	6	0.7446
Vermont	1	0.6072
Virginia	10	0.7227
Washington	1	0.435
West Virginia	9	0.2469
Wisconsin	12	0.7528
Wyoming	2	0.4123

## Discussion

Through simply probing social interest in COVID-19 vaccine-related queries, several meaningful relationships were delineated that have short and long-term implications on public health, vaccine rollout, and public sentiment analysis. Arguably, one of the first key steps taken in drawing meaningful relationships from social interest is identifying the common search queries that best encompass the context surrounding the searched event. To this end, our sensitivity syntax analysis enabled by GT allowed for the identification of temporal and regional trends with sustained and geographically ubiquitous interest. During correlation analysis, the most encompassing term leads to the highest correlation. Relative to the vaccination campaign, we observed the GT-produced related searches following a commitment correlation continuum, with each search phrase having varying degrees of commitment towards vaccination. In practice, it may be optimal to consider the degree of commitment in each search phrase to assess which geographic regions are failing to continue forward in the search progression (i.e., searching for “*how to get covid vaccine*” never progresses to “*covid vaccine appointment*”), which would indicate a failure in progression from an interest in obtaining the vaccine to formally scheduling a vaccination appointment. Additionally, combinatorial analyses with some or all of the related search phrases may best encompass the broadest trend while analyses with a single search phrase may enable practitioners to filter out extraneous information associated with unrelated events that may be driving changes in RSV.

To illustrate how more sensitive syntax can offer insight and context into more specific trends, we queried search interest in vaccines by manufacturer as well as interest in manufacturer-specific vaccine side effects. Not surprisingly, interest in vaccines appeared to be largely based on dose availability, with Pfizer and Moderna having the highest RSV, though as JNJ’s vaccine became readily available, its RSV surpassed that of Pfizer and Moderna. Interestingly, when the same search was carried out to include syntax related to side-effect interest, nearly all states (except MT, ND, TN, NC, and GA) had a higher interest in side effects related to Moderna, despite a similar prevalence of side effects compared to Pfizer’s vaccine. One explanation for the negative sentiment unjustifiably associated with Moderna’s vaccine is brand recognition [25]. We hypothesize that since Pfizer and JNJ have existed for much longer than Moderna and have a wide range of pharmaceuticals on the market, they have established recognizable brand imagery and trust. Confronting unjustified negative sentiment around a potentially life-saving vaccine through increasing dissemination of scientific information to the public may improve vaccination rates and establish brand trust in emerging pharmaceutical companies.

In addition to probing sentiment and relative interest in vaccine-related terms, we propose utilizing RSV to predict participation in vaccination. We demonstrated a broad correlation between RSV and daily vaccinations that, in practice, represents an easily implemented metric that can improve dose allocation and help better prepare healthcare facilities and regions for broader trends in vaccine participation. When estimating the amount of vaccines being administered, the public data does not differentiate between doses that are not yet used versus thrown away/wasted, though we broadly regard utilization as the percent of doses administered. Notably, we see the southeastern region of the US utilizing a relatively low percent of allocated doses. Interestingly, in several southern states (AL, GA, and KY) with low dose administration rates, a sharp divergence of RSV and daily vaccinations emerges. We postulate that the divergence in search interest may represent several scenarios. In one scenario, it may represent a natural decrease in interest, as the majority of the vaccine-eligible population has achieved vaccination and would no longer have an interest in obtaining a vaccine. In another scenario, it may represent a point of inflection in public sentiment, where the fraction of the population seeking vaccination is no longer increasing. In states with low vaccine utilization, this may indicate that increased efforts towards communicability and encouragement are warranted, particularly in rural areas with potential logistical limitations. Regardless of the catalyst behind a decreasing RSV in vaccine-related searches, sharp increases or decreases almost always correspond with changes in daily vaccination numbers; thus, probing interest offers a window of opportunity to adjust staffing and vaccine resources to best accommodate vaccine roll-outs.

Perhaps the most key finding of this work lies in the correlation between RSV and realized daily vaccination numbers. As one would expect, when interest increases or decreases in vaccine-related searches, a related increase/decrease tends to follow in daily vaccinations. This trend was observed in nearly every state and along the entire vaccination timeline. While this correlation alone is a useful tool for planning a large-scale or even local vaccination campaign, the implementation of lag correlation analyses further enhanced the significance of the predictive capability of social data. By identifying optimal lags (points at which RSV was most correlated with daily vaccinations), we could improve the significance of correlation and identify optimal predictive windows, which agencies may use to predict changes in vaccination trends. For example, states with high optimal lags would have several days to weeks to best prepare for a forthcoming trend. To this end, many states showed significant improvement in correlation when a lag was applied. These improvements in lag across a point in the 14-day temporal window represent both possible strengths and shortcomings in vaccine rollout. A shorter lag representing an improvement in Pearson correlation may indicate an optimally performing vaccination program where citizens are not required to wait. Negatively, these low lag values might show a population not voluntarily participating in the program, and a short window represents a bolus amount of vaccine that is readily available and not being utilized. On the other hand, if the optimal lag happens later in the window, it may represent shortcomings where vaccines are not readily available to those seeking them. Positively, a long lag might represent full enrollment as the states move through their tier systems and slowly enroll more groups.

Limitations 

This study has several potentially significant limitations. The analyses are prone to the natural limitations of the correlational research designs, as well as inherent instability in the rollouts of the various vaccination programs nationwide. One limitation is the usage of tiered vaccination systems (i.e., certain age brackets, pre-existing conditions, other vaccination order hierarchies) utilized by many states and the inability to quantify these impacts on the RSV data collected from Google. RSV may fluctuate with the localized interest of the population, and extensive wait times may be required by even the most enthusiastic of populations. Furthermore, a poorly designed tier progression may negatively impact the analysis of dosage utilization within each state. Overall availability can impact utilization as well, for even a state with an adequate amount of total vaccination sites and requisite staff may rapidly saturate their current tier and cause an artificial dip in daily vaccinations administered.

Applying the analysis to different cohorts at the state level fails to address the potential imbalances and limitations of vaccine availability in areas of varying population density, particularly between rural and urban communities. Rural populations that are seeking a particular brand of vaccination may be required to travel large distances or be simply unable to acquire their vaccine of preference. Furthermore, public perceptions of different vaccines caused by various sources, including media campaigns, can dramatically alter the trajectory of both RSV and daily vaccinations.

Each state employed a different timeline to appropriately budget and allocate vaccination doses across all of the primary suppliers. Consequently, the total JNJ vaccine was pulled from utilization due to fear of blood clots. These media campaigns add volatility and residual noise to the underlying trends being analyzed.

The final major limitation is the ubiquity of using a single search term across all states. A larger and more complete data set could be achieved by using aggregate search terminology to create composite relative search volumes. This method may yield a more robust data set with less overall variation across the temporal interval of study; however, this would also create a layer of obfuscation in the analysis, causing a further breakdown in the sensitivity of the correlation analysis and cross-correlations carried out. Further research would be required to accurately understand the different relationships when using aggregate data versus singular search terms.

## Conclusions

The findings from this study warrant the use of social data as an observational tool and an important metric in gauging voluntary vaccine participation. Relatedly, we demonstrate that search syntax can illustrate commitment to vaccination on a continuum. During dose allocation and staffing, it may be useful for state and local administrators to periodically observe public interest in the aforementioned search terms. Additionally, this study identifies an inflection point in public interest towards vaccination. When RSV in a search term supporting vaccination diverges from daily vaccinations and trends negatively, it may represent a plateau in those seeking vaccination and present a window of opportunity to further engage regions with decreasing interest and low vaccination rates. By understanding how the public as a whole engages in search querying, states and local governments can identify emerging hesitancy and appropriately address it through public discourse and policy. As scientists, it may be worthwhile to assess sentiment surrounding a search (i.e., inequity in searches for “vaccine side effects” by manufacturer) to increase scientific communication to the public to correct persisting disinformation.

Perhaps most importantly, we demonstrated that even a single search phrase regarding a large-scale event (i.e., vaccine roll-out) can encompass sentiment that directly correlates to public behaviors. In addition to using RSV to predict temporal and regional trends in vaccination behaviors, we identified optimal lag windows for best-fit prediction timeframes. The implementation of social data analyses during vaccine roll-outs has long-reaching impacts that may be applied well beyond the COVID-19 pandemic. In the future, a similar analysis can be conducted to improve dose allocations for vaccinations in emerging infectious diseases, which can reduce wait times, trim costs, reduce waste, identify under-vaccinated regions, and monitor changing sentiments during ever-changing public health crises.

## Figures and Tables

**Figure 6:**
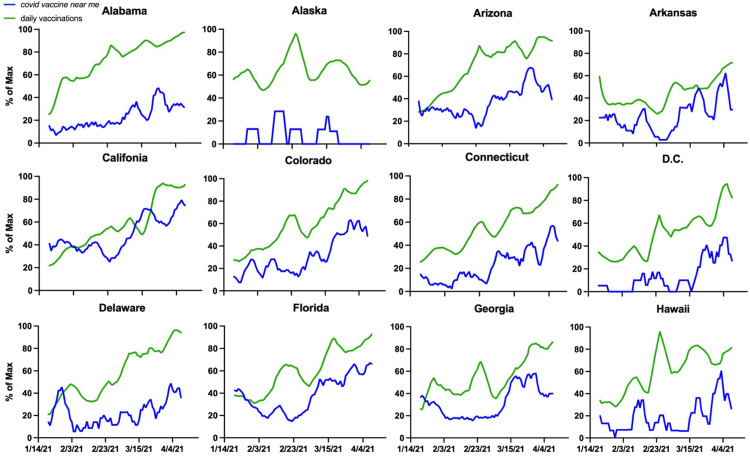
State-level temporal correlation between RSV for “covid vaccine near me” and daily vaccination numbers in states AL-HI RSV: relative search volume Data source: Google Trends and CDC VTrCK

**Figure 7:**
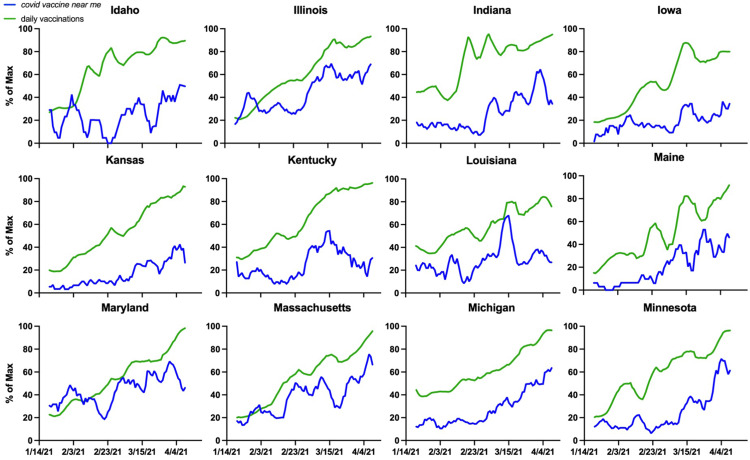
State-level temporal correlation between RSV for “covid vaccine near me” and daily vaccination numbers in states ID-MN RSV: relative search volume Data source: Google Trends and CDC VTrCK

**Figure 8:**
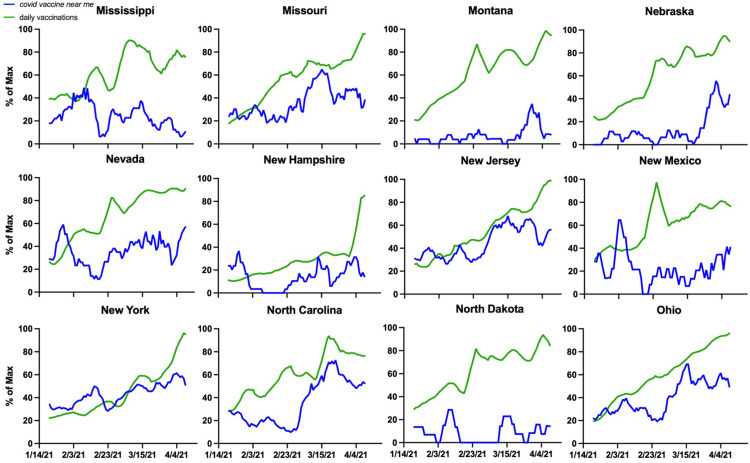
State-level temporal correlation between RSV for “covid vaccine near me” and daily vaccination numbers in states MS-OH RSV: relative search volume Data source: Google Trends and CDC VTrCK

**Figure 9:**
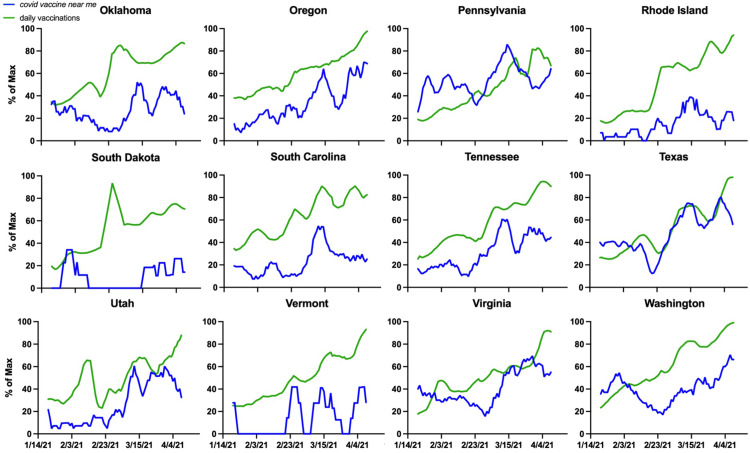
State-level temporal correlation between RSV for “covid vaccine near me” and daily vaccination numbers in states OK-WA RSV: relative search volume Data source: Google Trends and CDC VTrCK

**Figure 10:**
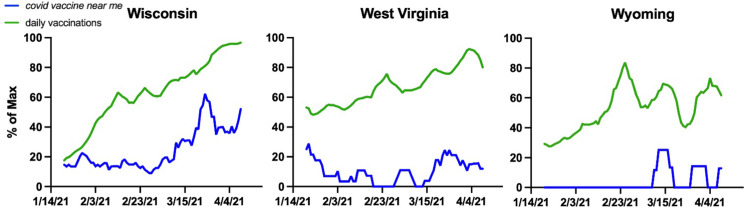
State-level temporal correlation between RSV for “covid vaccine near me” and daily vaccination numbers in states WI-WY RSV: relative search volume Data source: Google Trends and CDC VTrCK
